# Lung recruitment by continuous negative extra-thoracic pressure support following one-lung ventilation: an experimental study

**DOI:** 10.3389/fphys.2023.1160731

**Published:** 2023-05-15

**Authors:** Álmos Schranc, John Diaper, Roberta Südy, Ferenc Peták, Walid Habre, Gergely Albu

**Affiliations:** ^1^ Unit for Anesthesiological Investigations, Department of Anesthesiology Pharmacology, Intensive Care and Emergency Medicine, University of Geneva, Geneva, Switzerland; ^2^ Department of Medical Physics and Informatics, Albert Szent-Györgyi Medical School, University of Szeged, Szeged, Hungary

**Keywords:** lung recruitment, one-lung ventilation, negative-pressure ventilation, respiratory support, continuous negative extra-thoracic pressure

## Abstract

Lung recruitment maneuvers following one-lung ventilation (OLV) increase the risk for the development of acute lung injury. The application of continuous negative extrathoracic pressure (CNEP) is gaining interest both in intubated and non-intubated patients. However, there is still a lack of knowledge on the ability of CNEP support to recruit whole lung atelectasis following OLV. We investigated the effects of CNEP following OLV on lung expansion, gas exchange, and hemodynamics. Ten pigs were anesthetized and mechanically ventilated with pressure-regulated volume control mode (PRVC; FiO_2_: 0.5, Fr: 30–35/min, VT: 7 mL/kg, PEEP: 5 cmH_2_O) for 1 hour, then baseline (BL) data for gas exchange (arterial partial pressure of oxygen, PaO_2_; and carbon dioxide, PaCO_2_), ventilation and hemodynamical parameters and lung aeration by electrical impedance tomography were recorded. Subsequently, an endobronchial blocker was inserted, and OLV was applied with a reduced VT of 5 mL/kg. Following a new set of measurements after 1 h of OLV, two-lung ventilation was re-established, combining PRVC (VT: 7 mL/kg) and CNEP (−15 cmH_2_O) without any hyperinflation maneuver and data collection was then repeated at 5 min and 1 h. Compared to OLV, significant increases in PaO_2_ (154.1 ± 13.3 vs. 173.8 ± 22.1) and decreases in PaCO_2_ (52.6 ± 11.7 vs. 40.3 ± 4.5 mmHg, *p* < 0.05 for both) were observed 5 minutes following initiation of CNEP, and these benefits in gas exchange remained after an hour of CNEP. Gradual improvements in lung aeration in the non-collapsed lung were also detected by electrical impedance tomography (*p* < 0.05) after 5 and 60 min of CNEP. Hemodynamics and ventilation parameters remained stable under CNEP. Application of CNEP in the presence of whole lung atelectasis proved to be efficient in improving gas exchange via recruiting the lung without excessive airway pressures. These benefits of combined CNEP and positive pressure ventilation may have particular value in relieving atelectasis in the postoperative period of surgical procedures requiring OLV.

## Introduction

One-lung ventilation (OLV) is increasingly used due to both the technical advancements allowing easy instrumentation for lung isolation and the increased availability of minimally invasive techniques for thoracic surgeries (Campos, 2005). However, OLV augments the risk for the development of acute lung injury as a consequence of excessive lung tissue distensions generated by high driving pressures in the ventilated lung (Marini, 2018). Furthermore, the non-ventilated lung is subjected to prolonged hypoxic pulmonary vasoconstriction (Gong et al., 2010), which exacerbates regional lung inflammation (Lin et al., 2015). More importantly, the recruitment maneuver of the previously non-ventilated lung may elevate the stress and strain in the alveolar walls (Gattinoni and Pesenti, 2005), thereby exaggerating the shear forces both in the atelectatic and adjacent aerated alveolar regions (Mead et al., 1970; Tremblay and Slutsky, 2006; Duggan and Kavanagh, 2007). Thus, avoiding the development of severe lung injury following OLV is of paramount importance in preventing postoperative respiratory complications.

Continuous negative extra-thoracic pressure (CNEP) is in part based on the principle of the negative pressure ventilation technique (traditionally known as the iron lung), with the application of a cuirass-shell to optimize the negative pressure exposure to the chest (Kinnear et al., 1988). The ventilator applies negative pressure onto the chest and thereby creates more physiological pressure conditions as opposed to positive pressure ventilation due to increased transpleural pressure. This supportive mode facilitates recruitment of atelectatic areas (Samuels and Southall, 1989) with less adverse hemodynamic effects than conventional positive-pressure ventilation (Exovent Development, 2021). The application of CNEP is becoming increasingly common both in intubated and non-intubated patients especially in cases when non-invasive positive pressure ventilation is either contraindicated or not tolerated by the patient, during respiratory management (Exovent Development, 2021). Despite the increasing evidence, there is still a lack of knowledge on the ability of CNEP support to recruit whole lung atelectasis, such as observed following OLV.

We aimed at characterizing the ability of CNEP to recruit atelectatic lung areas both in the ventilated and non-ventilated lungs following OLV. We hypothesized that CNEP combined with conventional ventilation facilitates the recruitment of the previously non-ventilated lung, improving lung aeration and gas exchange following OLV. In addition, this benefit can be achieved without exerting excessive driving pressure at the airway opening.

## Materials and methods

### Ethics

The experimental protocol was approved by the Animal Welfare Committee of the Canton of Geneva and the Experimental Committee of the University of Geneva, Switzerland (no. 33212/GE30A, 16 February 2021). All procedures were performed in accordance with current Swiss animal protection laws (LPA, RS455). The current report follows the Animal Research: Reporting of *In Vivo* Experiments (ARRIVE) guidelines (Percie du Sert et al., 2020). Ten large-white, female pigs (45.5 ± 0.9 kg) were purchased from the farm of the University supplier (Markus Stirnimann, Apples, VD, Switzerland) and were delivered at least 3 days before the experiments to allow acclimatization. The pigs had access to food and water *ad libitum* before the experiments. In accordance with the 3R principles and the accepted ethics, the animals were used for additional investigations before this study protocol. The experiments were performed between 31 August and 11 September 2021.

### Animals and preparations

The animals were premedicated by intramuscular azaperone (8 mg/kg), midazolam (0.75 mg/kg) and atropine (25 μg/kg). Thirty minutes later, the animals were subjected to inhalation induction of anesthesia by sevoflurane (up to 6% end-tidal concentration), and an ear-vein was cannulated (22G Abbocath, Abbott Medical, Baar/Zug, Switzerland). Animals then received fentanyl (2 μg/kg) and atracurium (0.5 mg/kg) before laryngoscopy and tracheal intubation was performed with a 5.5 mm ID cuffed tube. Maintenance of anesthesia was achieved by iv infusion of propofol (10–15 mg·kg^-1^·h^-1^), fentanyl (10 μg·kg^-1^·h^-1^) and midazolam (0.1 mg·kg^-1^·h^-1^). After ensuring adequate levels of anesthesia and analgesia, atracurium was administered (1 mg·kg^-1^·h^-1^) to provide neuromuscular blockade. Pigs were ventilated with pressure-regulated volume control (PRVC) mode using a tidal volume (VT) of 7 mL/kg, a respiratory rate (RR) of 30–35/min, a fraction of inspired oxygen (FiO_2_) of 0.4, and a positive end-expiratory pressure (PEEP) of 5 cmH_2_O (Servo-I, Maquet Critical Care, Solna, Sweden) in the supine position. Setting of the respiratory parameters were based on a previous study using an identical animal model (Schranc et al., 2023). Femoral artery and jugular vein were cannulated for continuous hemodynamic measurements and blood sample withdrawal. Body temperature was measured with a rectal thermometer (Thermalert TH-8, Physitemp, Clifton, NJ, _USA_) and maintained at 38°C ± 0.5 °C using a heating pad (Mio Star, Zurich, Switzerland).

### Ventilation and hemodynamic monitoring

Tracheal pressure, heart rate and electrocardiogram (ECG) were recorded by PowerLab (PowerLab, ADinstruments, Oxfordshire, UK). Mean arterial pressure (MAP), cardiac output (CO) and extravascular lung water (EVLW) were determined by pulse index continuous cardiac output (PiCCO, PiCCO Plus, Pulsion Medical Systems, Munich, Germany) (Oren-Grinberg, 2010; Babik et al., 2017). Driving pressure was determined as the difference between the peak inspiratory pressure and PEEP. Furthermore, respiratory system elastance (E_RS_) was calculated as the difference between the plateau pressure and the PEEP divided by the VT. End-tidal CO_2_ concentration (ETCO_2_) and physiological dead space (Vd/VT) were determined by FluxMed monitor (MBMED, Buenos Aires, Argentina).

### Assessment of gas exchange

Arterial and venous blood samples were collected simultaneously to assess arterial partial pressure of oxygen (PaO_2_), carbon dioxide (PaCO_2_), and the central venous oxygen saturation (ScvO_2_) (VetScan i-STAT1, Abaxis, Union City, CA, United States). The PaO2/FiO_2_ ratio was also determined. The calculated capillary (CcO_2_), arterial (CaO_2_) and venous (CvO_2_) oxygen contents were used to determine the intrapulmonary shunt fraction (Qs/Qt) by applying the modified Berggren equation (Berggren, 1964; Wagner, 2015). Since the collection of mixed venous blood sample from the pulmonary artery requires highly invasive instrumentation, central venous blood was used for the assessment of intrapulmonary shunt.

$$\frac{Q_{s}}{Q_{t}}=\frac{{CcO}_{2}-{CaO}_{2}}{{CcO}_{2}-{CvO}_{2}}$$

### Estimation of lung aeration

Lung aeration was determined by electrical impedance tomography (EIT) in accordance with the international consensus statement (Frerichs et al., 2017). Briefly, an electrode belt containing 16 electrodes was placed around the chest at the fifth intercostal space and connected to a data acquisition unit (PulmoVista 500, Draeger, Lubeck, Germany).

EIT images of 32x32 pixels were constructed by the injection of small electrical currents (5 mA/50 Hz) using the manufacturer’s algorithm (Jang et al., 2019; Hahn et al., 2020). To assess lung aeration, end-inspiratory impedance values were assessed at three time points during the 2-minute-long recordings and ensemble averaged under each experimental condition. Global impedance data were extracted from these data sets and four regions of interest, defined as quadrants, were analyzed as the percentage of the global impedance values.

### Study protocol

**FIGURE 1:**
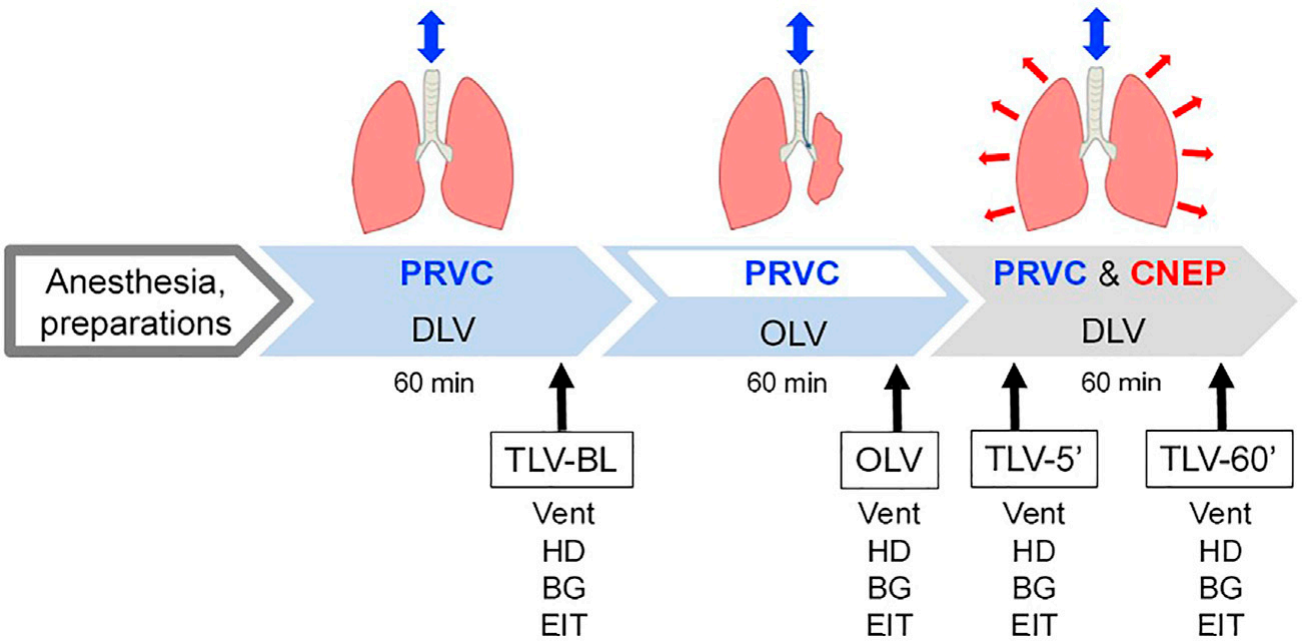
Schematic representation of the experimental protocol. PRVC, pressure-regulated volume control ventilation; TLV: two-lung ventilation; OLV: one-lung ventilation; CNEP: continuous negative extra-thoracic pressure. Measurements were performed under baseline condition during PRVC ventilation of both lungs (TLV-BL), 60 min after initiating one-lung ventilation (OLV) and five and 60 min after re-establishing two-lung ventilation with PRVC and CNEP support (TLV-5′ and TLV-60′, respectively). Vent: recording of ventilation parameters; HD: registration of hemodynamical variables; BG: blood gas analysis; EIT: electrical impedance tomography.

**FIGURE 2:**
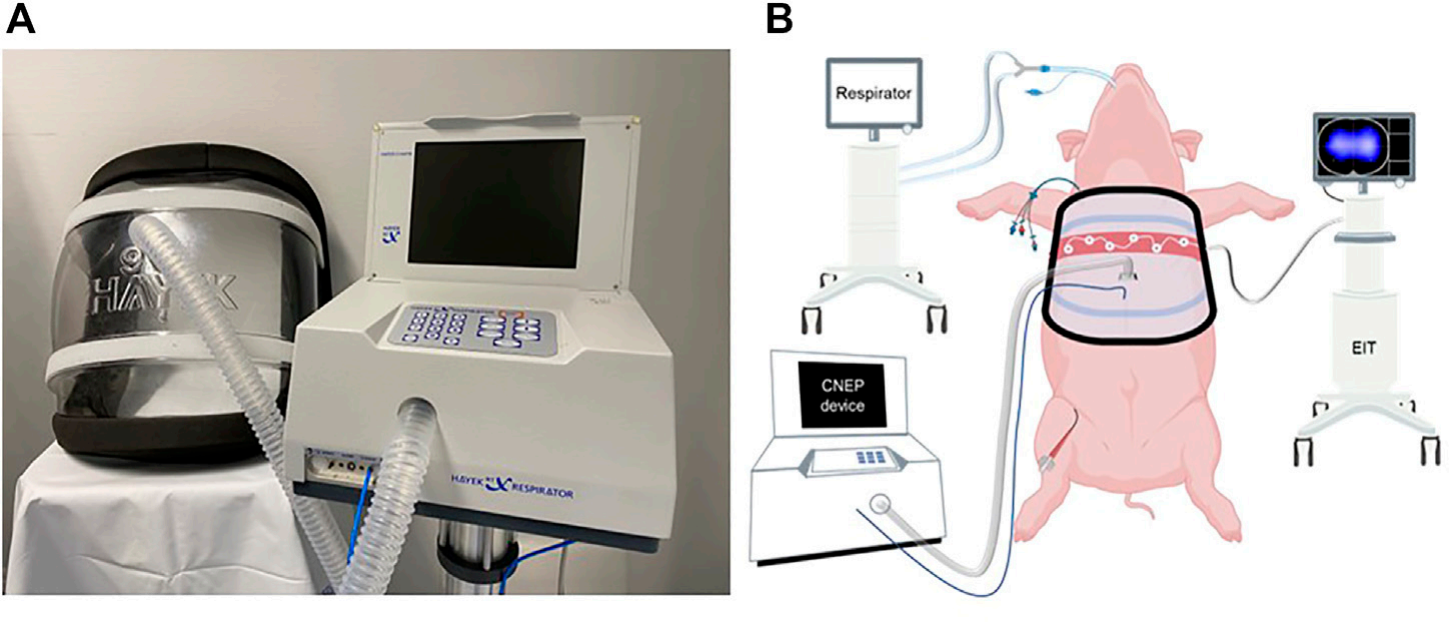
The Hayek RTX respirator **(A)** and the schematic of the measurement setup **(B)**.

The study protocol is presented in Figure 1. After anesthesia induction, instrumentation and surgical preparations, animals were ventilated with PRVC mode for 1-h, and a set of baseline data were collected (TLV-BL). To initiate OLV, lung isolation was subsequently ensured with the insertion of an endobronchial blocker (5.0 F, Arndt endobronchial blocker, Cook Medical LLC, Bloomington, IN, _USA_) under flexible bronchoscope guidance (Lohser and Slinger, 2015). Due to the presence of an accessory lobe, the complete isolation of the right lung is not feasible, therefore, the left lung was isolated in each animal. To mimic a clinical scenario, the body position of the pigs was changed by turning them to the right (30°). VT was then reduced to 5 mL/kg, and data collection was repeated after 1 hour of OLV (Schranc et al., 2023). The cuff of the blocker was deflated afterwards, and the ventilation of both lungs continued with a VT of 7 mL/kg and a CNEP support of −15 cmH_2_O (Hayek RTX, United Hayek Industries, London, United Kingdom) without applying any recruitment maneuver. Measurements were performed after 5 min (TLV-5′) and 1 h (TLV-60’) of PRVC supported by CNEP. At the end of the protocol, animals were euthanized by a single i. v. injection of sodium pentobarbital (200 mg/kg). The Hayek RTX respirator and the scheme of the measurement setup are presented in Figure 2.

### Exclusion criteria

All the experimental animals were included in the final data analysis.

### Sample size estimation

Since changes in alveolar ventilation would result in alterations in the elastic properties of the respiratory system, we used respiratory system elastance as the primary outcome variable to estimate the sample size for two-way repeated measures ANOVA, based on previous experimental outcomes obtained under similar experimental conditions (Fodor et al., 2019). A difference of 20% in respiratory system elastance between the ventilation modalities was considered as clinically relevant based on earlier publications using similar methodologies. We assumed a coefficient of variation of 10%, based on previously published data on respiratory system elastance that showed an approximately 10% coefficient of variation under similar experimental conditions (Petak et al., 2006). This analysis resulted in the need of at least nine animals to detect statistically significant changes with a statistical power of 0.9 and a two-sided alpha error of 0.05. Considering the potential drop-out rate of approximately 10%, we included 10 animals.

### Statistical analyses

Data are expressed as mean and standard deviation (SD). The Shapiro-Wilk test was used to test normality. One-way repeated measures ANOVA with Holm–Šidák post-hoc analyses were applied to test the differences between the different stages of the study protocol. The statistical tests were performed with SigmaPlot software package (Version 13, Systat Software, Inc., Chicago, IL, United States). Statistical analyses were conducted with a significance level of *p* < 0.05.

## Results

Testing the interval data and normality with Kolmogorov-Smirnov analysis and the homoscedasticity by Brown-Forsythe analysis, no significant difference was observed, therefore, all data for the measured variables fulfilled the assumptions of one-way repeated measures ANOVA.

**FIGURE 3:**
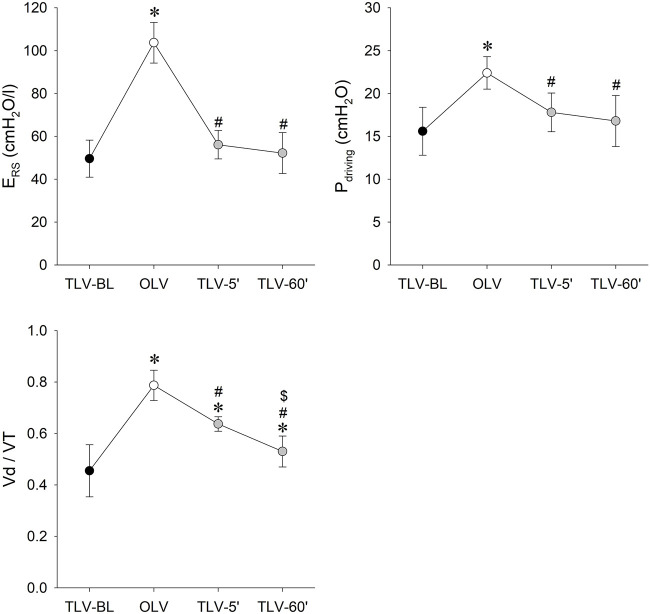
Measured and calculated ventilation parameters representing respiratory tissue stiffness (respiratory elastance: E_RS_), driving pressure (P_driving_) and physiological dead-space (Vd/VT) during the different protocol stages. Data are expressed as mean and SD. Black symbols: two-lung ventilation with PRVC mode under the baseline conditions (TLV-BL); white symbols: one-lung ventilation with PRVC mode (TLV-BL); grey symbols: re-established two-lung ventilation with PRVC mode and CNEP support for 5 (TLV-5′) and 60 min (TLV-60′). *: *p* < 0.05 vs. TLV-BL; #: *p* < 0.05 vs. OLV; $: *p* < 0.05 vs. TLV-5’.

Ventilation parameters are summarized in Figure 3 for the different phases of the protocol. Compared to all conditions with two-lung ventilation, significantly higher E_RS_ and P_driving_ was evidenced during OLV (*p* < 0.001 for all). Parameters E_RS_ and P_driving_ returned to their baseline levels already at 5 min and remained at this level following 60 min of CNEP support. The Vd/VT increased significantly during OLV (*p* < 0.001); and recovered gradually after applying CNEP ventilation support for 5 min (TLV-5′, *p* < 0.001) and 60 min (TLV-60’, *p* = 0.019).

**FIGURE 4:**
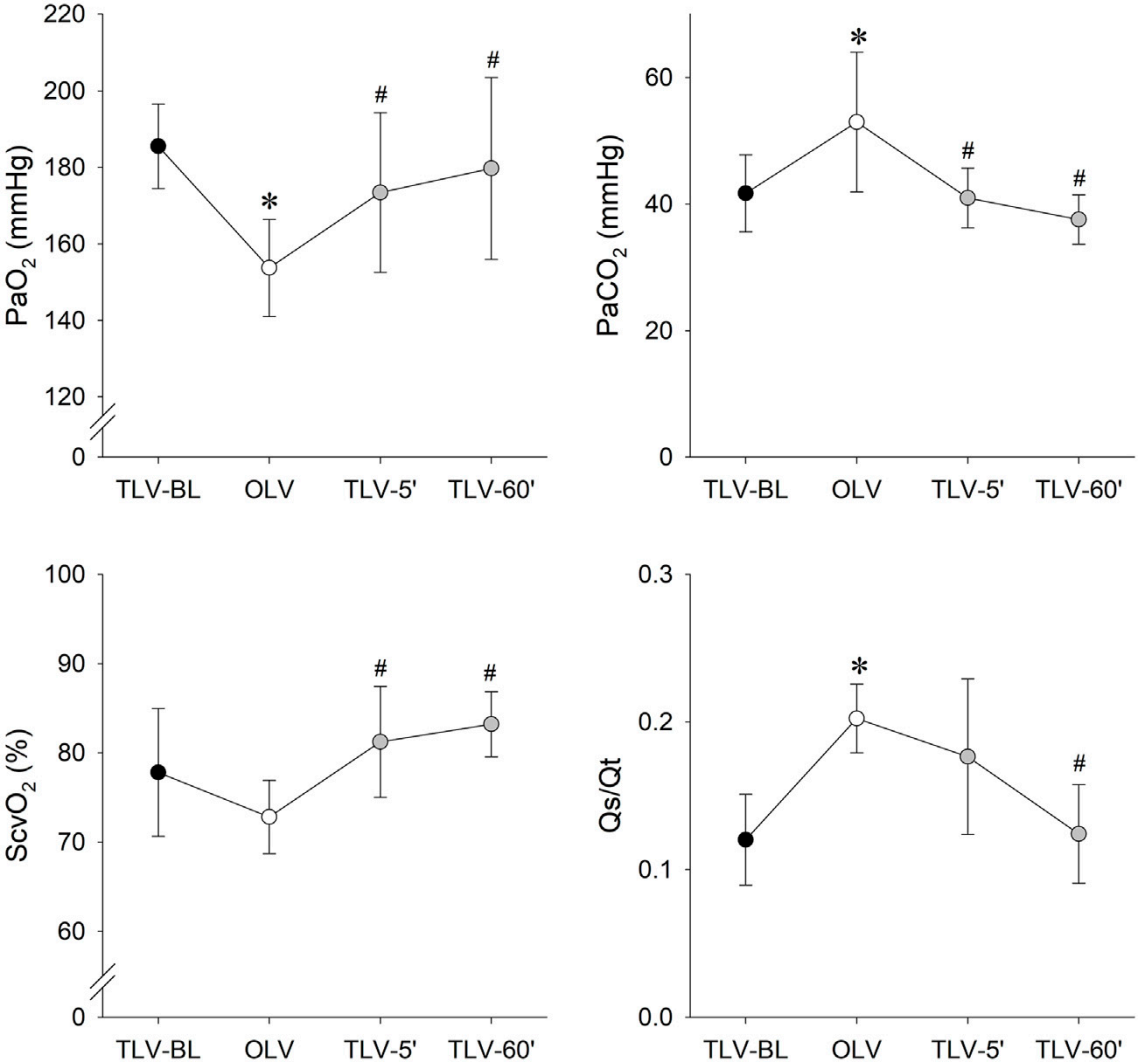
Gas-exchange parameters obtained during the different protocol stages. Data are expressed as mean and SD. Black symbols: two-lung ventilation with PRVC mode under the baseline conditions (TLV-BL); white symbols: one-lung ventilation with PRVC mode (TLV-BL); grey symbols: re-established two-lung ventilation with PRVC mode and CNEP support for 5 (TLV-5′) and 60 min (TLV-60′). PaO_2_: arterial partial pressure of oxygen; PaCO_2_: arterial partial pressure of carbon dioxide; ScvO_2_: central venous oxygen saturation; Qs/Qt: intrapulmonary shunt fraction. *: *p* < 0.05 vs. TLV-BL; #: *p* < 0.05 vs. OLV.

Figure 4 shows the gas exchange parameters during the different stages of the study protocol. While PaO_2_ was significantly lower during OLV compared to TLV-BL (*p* < 0.001), it increased significantly in TLV-5’ (*p* = 0.05) and TLV-60’ (*p* < 0.001) reaching the levels obtained under the baseline conditions. These changes were in accordance with the elevated PaCO_2_ level during OLV compared to TLV-BL and the diminishments during TLV-5′ and TLV-60’ (*p* < 0.001 for all). Elevation of ScvO_2_ were observed at TLV-5’ (*p* = 0.008) and TLV-60’ (*p* < 0.001) compared to those obtained during OLV. Qs/Qt elevated significantly during OLV (*p* = 0.028) and returned gradually to the baseline level during CNEP support (*p* = 0.025 between TLV-5′ and TLV-60’).

**FIGURE 5:**
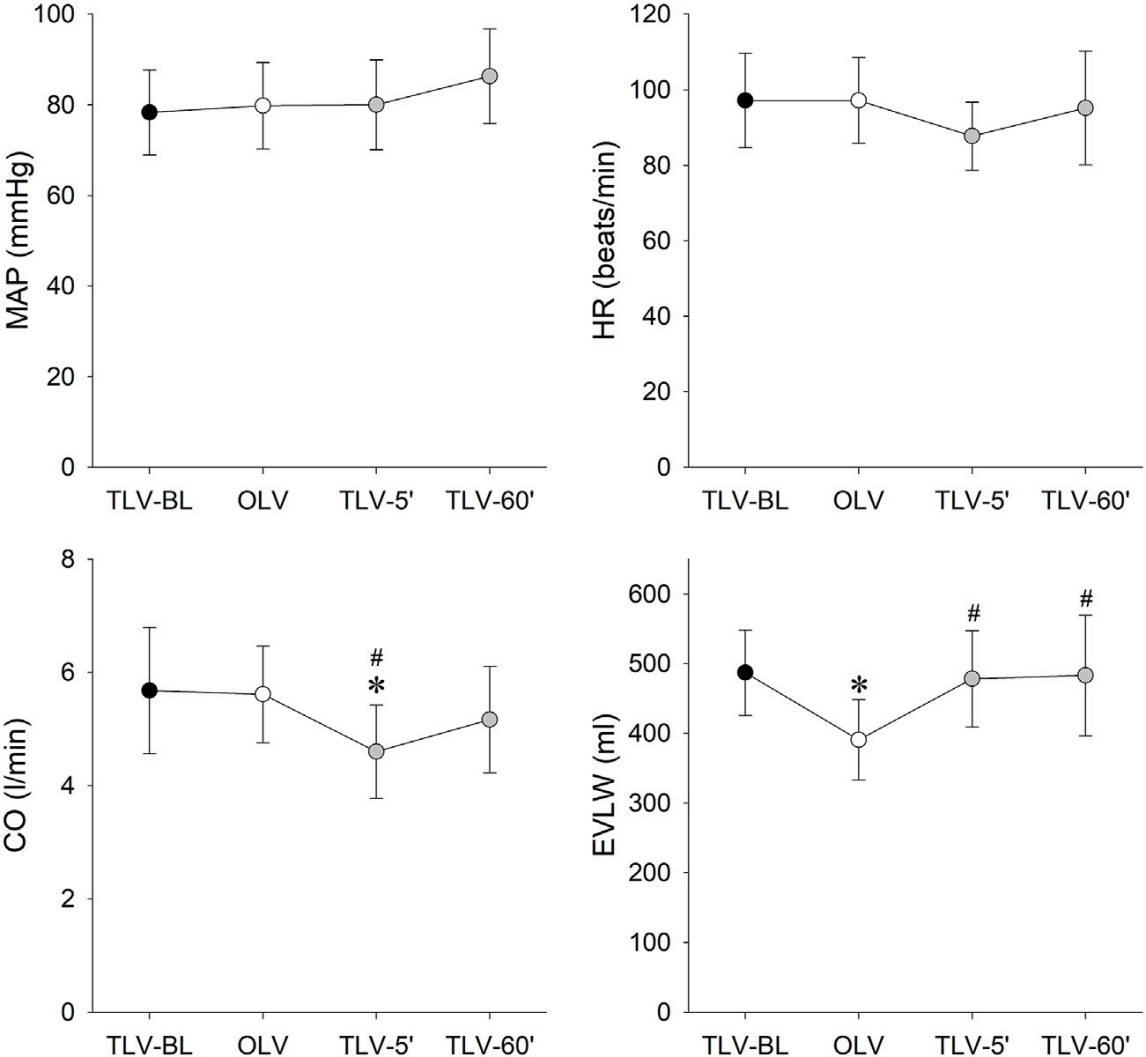
Changes in hemodynamic parameters during two-lung ventilation with PRVC mode under the baseline conditions (TLV-BL, black symbols), one-lung ventilation with PRVC mode (TLV-BL, white symbols) and 5 (TLV-5′) and 60 min (TLV-60′) after re-establishment of two-lung ventilation with PRVC mode and CNEP support. Data are expressed as mean and SD. MAP: mean arterial pressure; HR: heart rate; CO: cardiac output; EVLW: extravascular lung water. *: *p* < 0.05 vs. TLV-BL; #: *p* < 0.05 vs. OLV.

Hemodynamic parameters obtained during the various interventions are depicted on Figure 5. OLV and CNEP support had no significant effects on MAP and HR. While significant drop in CO was observed 5 min after CNEP support (TLV-5′) compared to BL (*p* = 0.02) and OLV (*p* = 0.026), this difference vanished by 60 min (TLV-60′). A significant decrease of EVLW was evidenced during OLV compared to TLV-BL (*p* = 0.008), EVLW returned to the baseline level and showed significant differences under TLV-5′ and TLV-60’ conditions compared to OLV only (*p* = 0.012 and *p* = 0.01, respectively).

**FIGURE 6:**
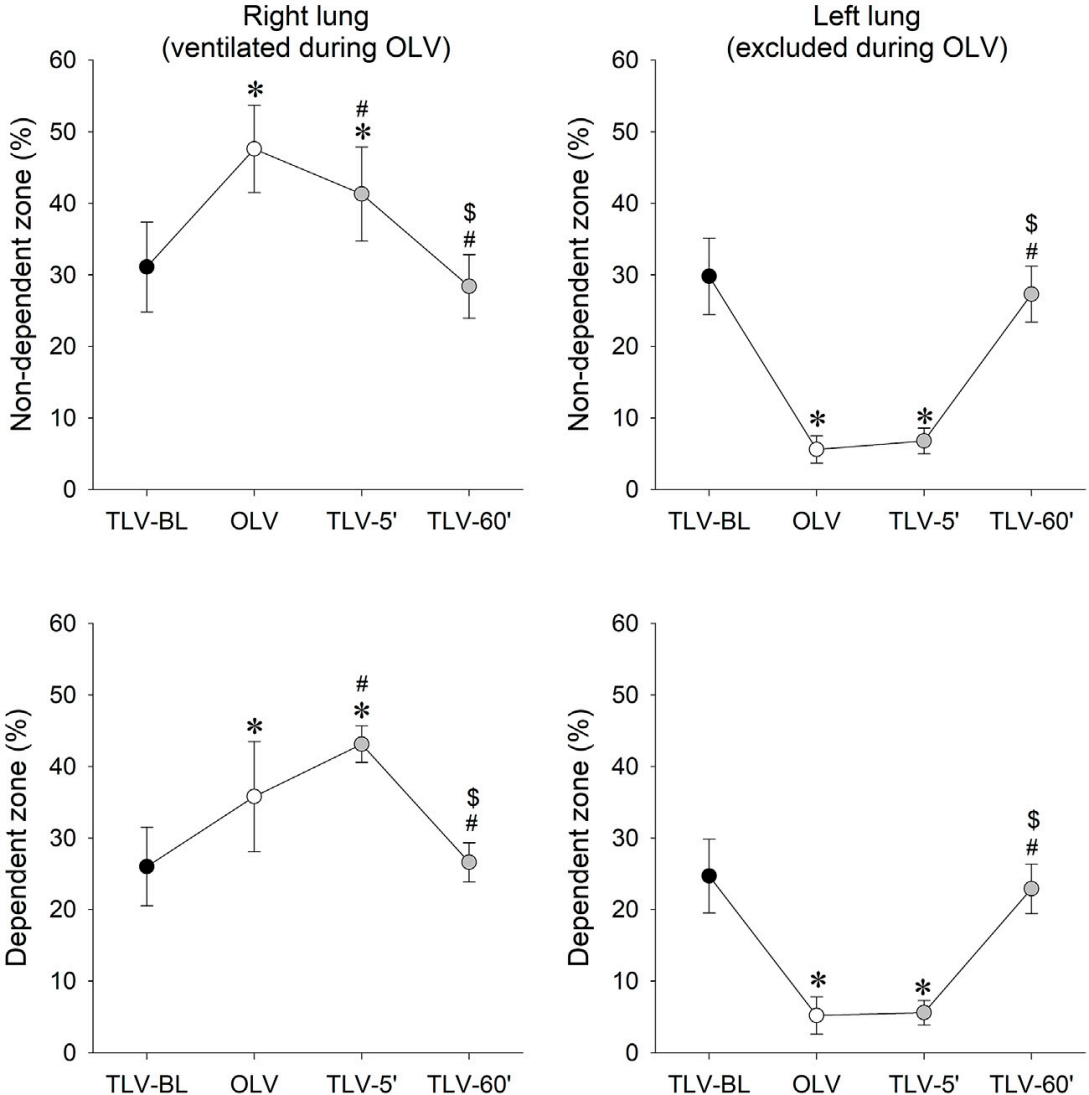
Relative contributions of each lung region to global electrical impedances in percentage, under baseline conditions when two-lung ventilation was performed with pressure-regulated volume control mode (TLV-BL), following one-lung ventilation (OLV), and 5 (TLV-5′) and 60 min (TLV-60′) following PRVC ventilation mode was supplemented with continuous negative extra-thoracic pressure (CNEP) support. Data are expressed as mean and SD. *: *p* < 0.05 vs. TLV-BL; #: *p* < 0.05 vs. OLV; $: *p* < 0.05 vs. TLV-5’.

Relative contributions of each lung region to global electrical impedance data are presented in Figure 6. As expected during OLV, aeration increased in the ventilated right lung and deteriorated in the non-ventilated left lung compared to TLV-BL (*p* < 0.001 for both). Five minutes after the onset of CNEP support, an improvement in aeration in the dependent dorsal zone was observed on the expense of a diminished aeration in the non-dependent ventral area (*p* < 0.001 for both zones). As concerns the left lung that was excluded during OLV, the lung remained poorly aerated 5 min after initiating CNEP support (TLV-5′) in both the dependent and non-dependent zones (*p* < 0.001 for both) but exhibited marked elevations 60 min after CNEP support (TLV-60’, *p* < 0.001 for both). Aeration of both lung sides and zones returned to the baseline condition (TLV-BL) after 60 min of CNEP support.

## Discussion

In the present study we investigated the ability of CNEP combined with conventional ventilation to recruit whole lung atelectasis following OLV. We compared gas exchange, ventilation and hemodynamic parameters along with lung aeration in an animal model of whole lung atelectasis. Data were collected following 1 hour of two-lung ventilation and 1 hour of OLV with PRVC, then finally 1 hour combining PRVC with CNEP in an attempt to re-expand the collapsed lung. Our experiment revealed that already after 5 min of CNEP support, gas exchange improved and E_RS_ and P_driving_ values were lowered. These beneficial changes in the respiratory outcomes were associated with an immediate augmented aeration of the dependent zone of the non-collapsed right lung. After an hour of supplemental CNEP, indices reflecting gas-exchange, hemodynamics and lung aeration returned to their respective values measured under the baseline conditions.

The major challenge during recruitment following OLV is to prevent lung injury in both atelectatic and aerated lung regions (Mead et al., 1970; Tremblay and Slutsky, 2006). Considering that acute lung injury and acute respiratory distress syndrome are the leading cause of morbidity and mortality after thoracic surgery, it is necessary to prevent the occurrence of ventilation-induced lung injury (Licker et al., 2009; Lohser and Slinger, 2015). Previous clinical studies demonstrated the beneficial effects of CNEP on gas-exchange and hemodynamic parameters in adult patients following thoracic surgery (Chaturvedi et al., 2008) and in children with mild respiratory disease or severe respiratory infections (Ishimori et al., 2021a; Ishimori et al., 2021b).

During routine clinical practice, different strategies have been proposed to re-expand the excluded lung following OLV. All methods are aimed at recruiting the lung while minimizing the alveolar stress and strain and preventing pulmonary edema induced by the ischemia-reperfusion encountered following thoracic surgery (Bender et al., 2018). The use of recruitment maneuvers is still routinely considered in this clinical scenario despite the potential risk of inducing lung damage (Lohser and Slinger, 2015). Previous results revealed differences in localized strain distribution between ventilation regimes demonstrating reduced local stretch and distortion during negative pressure ventilation compared to the conventional positive pressure lung expansions (Sattari et al., 2023). More importantly, another study concluded that parenchymal strain should not exceed the threshold level of repair mechanisms to avoid lung injury (Protti et al., 2011). Therefore, the combination of positive and negative pressure ventilation may avoid the overcome of strain-stress on the critical threshold level, thereby recruiting the lung without inducing lung tissue damage. Alternatively, variable ventilation has been proposed to re-expand the lung without the need for recruitment maneuvers (Mutch et al., 2000). Nevertheless, the benefit of variable ventilation on lung recruitment is at the expense of a need for a periodic elevation of driving pressure (Ma et al., 2011). It is important to note that in the present study, the complete recovery of all respiratory outcomes was achieved without elevating driving pressure (Figure 3). This benefit of CNEP may be attributed to the more efficient effect of negative extrathoracic pressure on the recruitment of pulmonary capillaries (Petak et al., 2009) and/or to the possible facilitation of alveolar expansion exerted by a more physiological transpulmonary pressure regimen (Bancalari et al., 1973). Furthermore, no elevation in EVLW was observed compared to the baseline condition at any stage when CNEP support was applied. Accordingly, it can be anticipated that the applied negative extrathoracic pressure support exerted its benefit without inducing excessive pulmonary oedema.

The primary findings of the present study are that after 5 min of CNEP support improved gas exchange was observed (Figure 4) with an elastance and driving pressure comparable to that measured at BL (Figure 3). Lung aeration in the non-ventilated, left lung achieved the physiological level only after 1 hour of CNEP application, however, the aeration in the dorsal zone of the right lung increased at the beginning of CNEP support (Figure 6). Hence, the improvement in gas-exchange after 5 min of CNEP is due to the improved aeration in the dependent zone of the right lung and not the recruitment of the left lung. Furthermore, the lack of difference in the Qs/Qt values between OLV and TLV-5’ is in accordance with the poor aeration observed in the left lung.

In the present experimental design, the effects of PRVC alone when re-establishing TLV was not assessed. This approach is in complete agreement with the 3R guidelines to minimize the number of animals involved in research. Although oxygenation improved 10 min and 1 hour following re-establishing TLV after OLV in an identical animal model, PaO_2_ did not reach the values measured at baseline (Mutch et al., 2000). Furthermore, the dynamics of the oxygenation improvement in the present study differs from earlier results without CNEP, as no recovery in PaO_2_ to the baseline was reported during the 5 h of TLV following OLV. Nevertheless, a complete recovery of all respiratory outcomes was observed in the present study. which can be attributed to the supplemental application of CNEP rather than spontaneous recovery.

Although OLV had no effect on CO, following the removal of the endobronchial blocker, due to the inflation of the left lung and the elevated intrathoracic pressure, a decreased CO was observed 5 min following onset of CNEP support. Another potential explanation for this transient drop in CO may be related to the remaining pulmonary hypoxic vasoconstriction and elevated pulmonary vascular resistance, which disappeared at 60 min with the total re-expansion of the left lung. In addition, CNEP did not affect MAP and HR that remained stable throughout the experiment. The positive circulatory effects of negative pressure ventilation, such as the increase of cardiac output is a well-known phenomenon and has been reported by previous studies (Kilburn and Sieker, 1960; Shekerdemian et al., 1999). The lack of changes in these parameters suggest that the positive inotropic and chronotropic effects of the negative extra-thoracic pressure were masked by the deleterious hemodynamic effects of the conventional positive-pressure ventilation. The decrease in EVLW during OLV is a known phenomenon, which may be explained by atelectasis induced hypoxic vasoconstriction (Kuzkov et al., 2007; Yasuuji et al., 2014) and/or the systematic underestimation of EVLW due to changes in the thermodilution curve (Haas et al., 2013). More importantly, EVLW returned to the baseline value when CNEP was applied and remained stable until the end of the experiment.

Previous studies described certain adverse events regarding the application of CNEP, such as hypothermia and skin lesions, predominantly in the pediatric population (The perils of paediatric research, 1999). Since in our protocol, conditions of body temperature were controlled, we did not observe hypothermia. Regarding the risk of skin lesions, our experimental subjects do not represent the same skin characteristics as neonates and children. However, the lack of skin lesions on the animals, is in accordance with the findings of recent clinical studies (Nunez and Hassinger, 2020; Ishimori et al., 2021a).

A few limitations relevant to the present study warrant consideration. Only female pigs were involved in our experiments; therefore, the study design does not allow for the assessment of possible sex-related differences. As the goal of our study was to investigate the ability of CNEP to recruit atelectatic lung without using any hyperinflation maneuver, there was no other study group to compare CNEP support with different recruitment maneuvers used in clinical practice. Since the cuirass of the respirator was designed for human use, another limitation could be the structural differences between the human and pig chest anatomy. Although our OLV model mimics the characteristics of a thoracic surgery setting, application of CNEP in the postoperative period could be limited due to the localization of surgical incisions (e.g., anterolateral thoracotomy) and chest drain placement.

In summary, the results of the present study demonstrated that the application of CNEP combined with a conventional positive-pressure ventilation modality proved to be efficient in lung recruitment following OLV without inducing excessive airway pressures. Moreover, the beneficial effect of CNEP could be potentially further optimized with other non-hyperinflation recruitment techniques by positive-pressure ventilation, such as an extended inspiratory duration. CNEP support improved gas-exchange already 5 minutes following OLV and at 1 hour, the aeration in the left lung returned to physiological range without the demand of excessive driving pressures. The lack of differences in elastance during CNEP support and BL measurements suggest that this combined ventilation proposes a protective alternative in recruitment of whole lung atelectasis where hyperinflation maneuvers can be deleterious to the lung parenchyma.

## Data Availability

The raw data supporting the conclusion of this article will be made available by the authors, without undue reservation.

## Abbreviations

ANOVA, analysis of variance; ARRIVE, animal research reporting of *in vivo* experiments; BL, baseline; CaO_2_, calculated arterial oxygen content; CcO_2_, calculated capillary oxygen content; CNEP, continuous negative extra-thoracic pressure; CO, cardiac output; CvO_2_, calculated venous oxygen content; ECG, electrocardiogram; EIT, electrical impedance tomography; E_RS_, elastance of the respiratory system; FiO_2_, fraction of inspired oxygen; G, respiratory tissue damping; H, respiratory tissue elastance; HR, heart rate; i.v, intravenous; I, E ratio: inspiratory-to-expiratory time ratio; MAP, mean arterial pressure; OLV, one-lung ventilation; PaCO_2_, arterial partial pressure of carbon dioxide; PaO_2_, arterial partial pressure of oxygen; PEEP, positive end-expiratory pressure; PiCCO, pulse index continuous cardiac output; PIP, peak inspiratory pressure; PRVC, pressure-regulated volume control ventilation; Qs/Qt, intrapulmonary shunt fraction; RR, respiratory rate; SD, standard deviation; ScvO_2_, central venous oxygen saturation; VT, tidal volume.
